# Dual effects of technology change: How does water technological progress affect China’s water consumption?

**DOI:** 10.1016/j.isci.2022.104629

**Published:** 2022-06-17

**Authors:** Xiaohui Yang, Wenwen Chen, Mingdong Jiang, Ping Jiang, Xiaomei Shen

**Affiliations:** 1 Department of Environmental Science and Engineering, Fudan Tyndall Centre, Fudan University, Shanghai 200433, China; 2 Business School, Hohai University, Nanjing 211100, China; 3 College of Environmental Sciences and Engineering, Peking University, Beijing 100871, China; 4 School of Environmental Science and Engineering, Yancheng Institute of Technology, Yancheng 224051, China; 5 Green, Low-carbon and Circular Economy Institute of Yancheng, Yancheng Institute of Technology, Yancheng 224051, China

**Keywords:** Natural resources, Environmental issues, Hydrology

## Abstract

Water technological progress contributes a lot to water conservation. Most studies have overestimated its contribution by ignoring its scale effect on economic growth, leading to the increase in water consumption. To quantify the trade-off of water technological progress, we combine the macroeconomic model with the environmental model to analyze both the scale effect and the intensity effect of water technological progress. Results show that the intensity effect has reduced China’s water consumption by 612.256 × 10^9^ m^3^ from 2003 to 2020, while the scale effect increases China’s water consumption by 189.911 × 10^9^ m^3^. The contribution of technological progress varies among regions in China. The industrial structure effect inhibits water consumption, second to the water-saving effect of water technological progress. The input effect increases water consumption owing to the particularly striking promotion of the effect of capital input. Some policy recommendations are given to mitigate the trade-off of water technological progress and regional disparity.

## Highlights

• The trade-off of water technological progress on water consumption is quantified
• Water technological progress reduces water consumption in general
• The scale effect of it offsets part of its water-saving effect
• The effect of water technological progress has obvious regional disparity

## Graphical abstract

**Figure:**
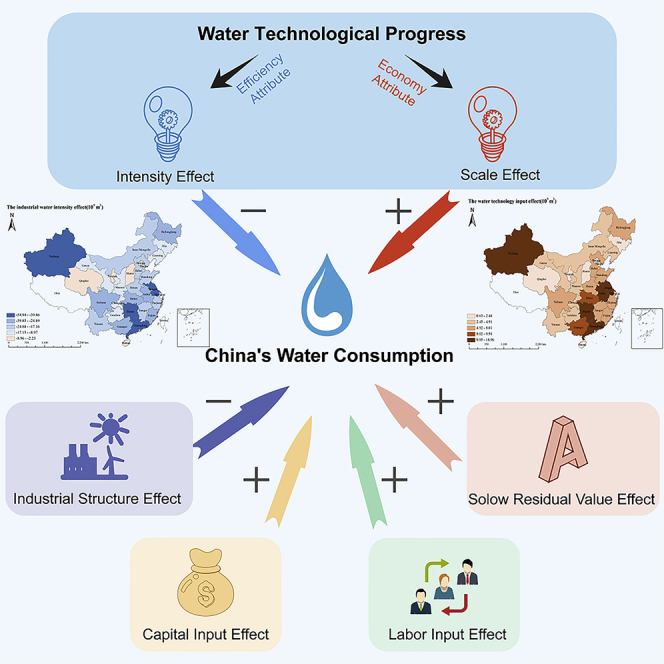

## Introduction

Water resources play a vital role in economic and social development (Jia et al., 2018). However, water shortage has become a major bottleneck, restricting the sustainable development of the global economy (Pokhrel et al., 2021). China is one of the countries which urgently lack water. Water availability per capita in China accounts for only about 1/4 of the world average, and the pressure of water consumption becomes increasingly prominent (Dong et al., 2014). The distribution of water resources shows a strong regional disparity in China. The vulnerability of water resources in the northern and central coasts of China is higher than the southwest region (Cai et al., 2017). The geographical mismatch between industrial water demand and water endowment poses great threat to sustainable water supplies in China (Yu, 2011). However, water resource exploitation is unequal in the face of increasing regional disparity, with high water deprivation in the western region (He et al., 2019). To alleviate water shortage and uneven distribution, China has made many efforts, including increasing the total water supply through water extraction, storage (Zhao et al., 2015), and desalination (Zheng et al., 2014) and reducing water scarcity and its inequality with water transfer projects (Sun et al., 2021). However, the current water supply management has not effectively solved the problem (Wang et al., 2015).

According to the International Patent Classification (IPC), water technologies encompass the innovation related to water supply, water distribution, treatment, and sewage, such as irrigation technologies, water collection and distribution, groundwater extraction, desalination, and so forth. (Moro et al., 2018). A significant number of studies have shown that water technological progress can contribute to water conservation by reducing the water intensity. For example, Wang and Wang (2020) found water technological progress drove the occurrence of decoupling between China’s water consumption and economic growth. Song et al. (2018) analyzed factors that affected water resource efficiency and proved the important role of technological improvement in promoting water efficiency. Zhang et al. (2019) pointed out that modern agricultural irrigation technologies have been identified as an important measure against water shortage. Scholars have also studied the regional disparity for this effect. Technological progress had a positive effect on the green total factor efficiency of industrial water resources in western China, while it had a negative effect in central China (Jin et al., 2019). Zou and Cong (2021) established an evaluation index system of water resource utilization efficiency and found technological progress improved the efficiency which was high in the eastern coastal region while was an undesirable level in central and western China.

However, the impact of technological progress is not unilateral. Li et al. (2022) found government mandatory energy-biased technological progress increased the amount of coal consumption. Liao and Ren (2020) found that when the level of technological progress was lower than a certain threshold, it positively impacted resource utilization efficiency, or vice-versa. In summary, technological progress has dual effects. Li and Wang (2017) argued that technology had relatively independent economic and environmental attributes, shown as intensity effect and scale effect on carbon emission. Similarly, it remains a question whether water technological progress has dual effects on water consumption. There was a paradox between the wide application of water-saving technologies and a more severe regional water shortage (Zhou et al., 2021). Taking western Kansas as an example, the shift to more efficient irrigation technology did not reduce groundwater extraction (Pfeiffer and Lin, 2014). How do dual effects of water technology change affect water consumption? With the continuous expansion of the depth and breadth of technology, the negative effects of modern technology have become more complex and hidden. On the one hand, the advancement of water technology reduces production costs (Judd and Carra, 2021) with fixed water price, which brings about the economic scale expansion under the market mechanism, thereby increasing the water resource consumption. On the other hand, the advancement of water technology will give birth to new products and industries (Hasanbeigi and Price, 2015), which will bring resource dependence and excessive consumption. In this article, we define the negative effect mentioned above as the scale effect of water technological progress.

To quantify the scale effect, the extended Cobb-Douglas (C-D) production function is introduced. The extended C-D production function can measure the contribution of water technological progress, capital growth, and labor growth to economic growth based on the tradition model (Yuan et al., 2009). Furthermore, the Kaya identity is the most important technique to illustrate the relationship between water consumption and various macroeconomic and source-related variables, such as GDP, water intensity, and industrial structure. The Logarithmic Mean Divisia Index (LMDI) method is widely applied in the driving factor analysis of energy consumption (Ang and Wang, 2015) and carbon emission (Ang and Goh, 2019). Long et al. (2019) and Zhang et al. (2020) applied this method to analyze driving factors of water consumption. Referring to existing studies, this article combines the extended C-D production function with the Kaya identity and further uses the LMDI model to decompose the driving factors of China’s water consumption from 2003 to 2020, including the scale effect and the intensity effect of water technological progress.

The contribution of this article is as follows: First, the article carries out the quantitative analysis of the actual contribution of water technological progress on water consumption reduction, which was overestimated in the previous studies, through a state-of-art framework developed by the study for the analysis of the dual effects of technological progress on water consumption. Second, the environmental model is combined with the macroeconomic model which is oriented by water technology to explore the impact mechanism of water technological progress. The macroeconomic model explores the impact of water technological progress on economic growth, and the environmental model analyzes its impact on water consumption by expanding the scale of production. Compared with the available literature, we break the limitation of the current model framework from the single-disciplinary perspective, thus the accuracy and comprehensiveness of analysis in our study can be significantly improved. Third, this article discusses the temporal and spatial difference in the water-saving effects of technological progress on water consumption, and based on the outcomes of the study, this article can give more practical recommendations on how to mitigate regional disparity in developing countries.

## Results

### China’s water consumption in three industries

**Figure 1:**
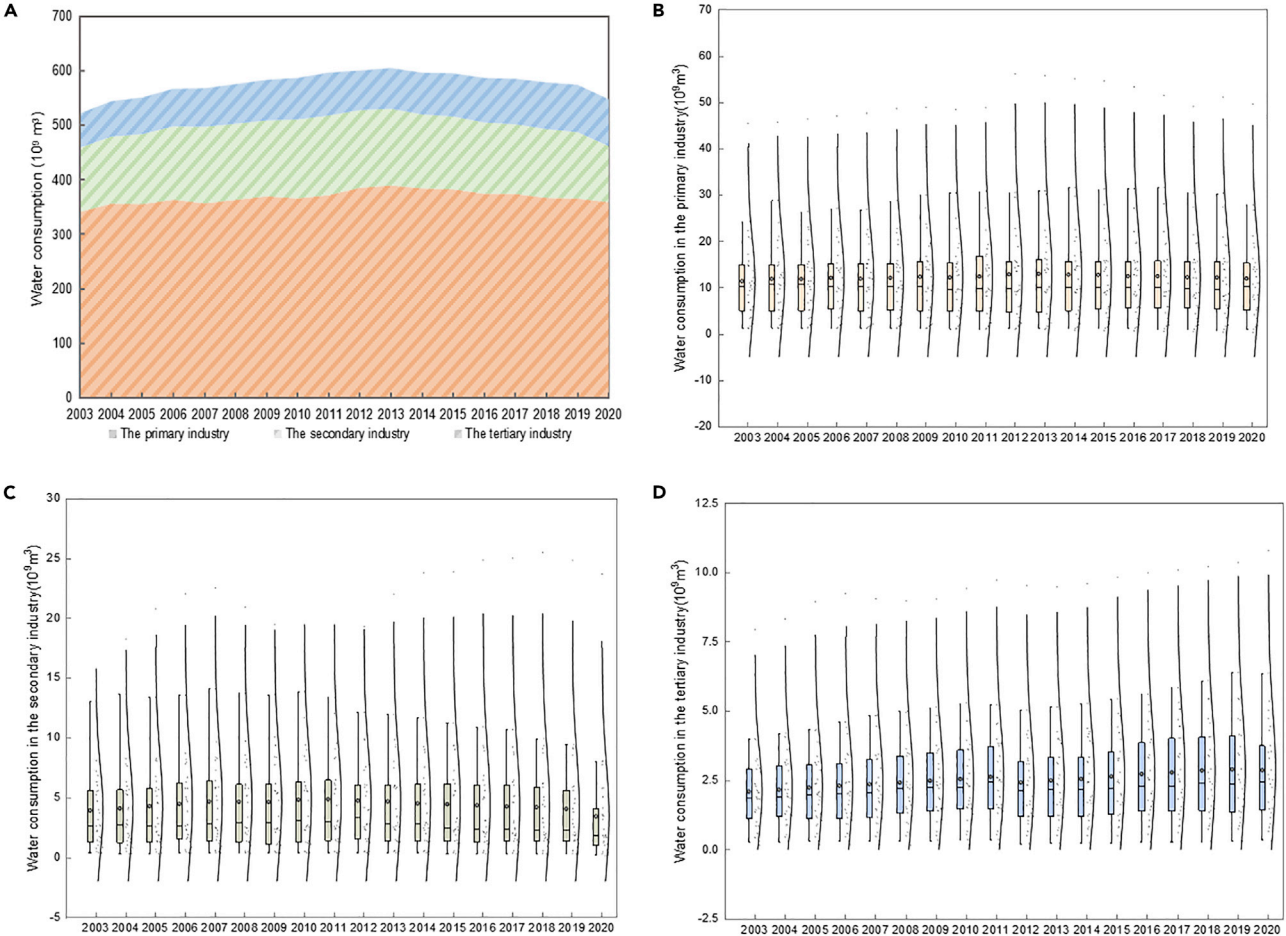
The trends of primary, secondary, and tertiary industry water consumption from 2003 to 2020 (A) Water consumption in three industries. (B) Water consumption in the primary industry. (C) Water consumption in the secondary industry. (D) Water consumption in the tertiary industry.

As shown in Figure 1A, the total water consumption in China showed an inverted U-shaped trend. From 2003 to 2013, the water consumption increased from 521.61 × 10^9^ m³ to 604.81 × 10^9^ m³, reaching its peak in 2013. After 2013, the water consumption began to decline and reduced to 547.44 × 10^9^ m³ in 2020. Total water consumption increased by 5% from 2003 to 2020. Agricultural water consumption accounted for 63.92% of the total water consumption, much higher than the water consumption of other industries.

The primary industry water consumption showed an inverted U-shaped trend (Figure 1B), increasing to a peak of 384.13 × 10^9^ m³ in 2014, and then falling back to 358.51 × 10^9^ m³ in 2020. The secondary industry water consumption also showed an inverted U-shaped trend (Figure 1C), increasing to a peak of 146.01 × 10^9^ m³ in 2011, and then falling back to 102.92 × 10^9^ m³ in 2020. The tertiary industry water consumption showed a monotonous increasing trend (Figure 1D), reaching the highest point of 86.88 × 10^9^ m³ in 2019. The median value of water consumption in the three industries was concentrated and the distribution of extreme values was sparse. The average value was generally higher than the median value, indicating that there were significant differences in water consumption among different provinces. Among three industries, the dispersion degree of the tertiary industry water consumption was higher, indicating that the region disparity in tertiary industry water consumption was particularly significant. For example, the tertiary industry water consumption in Qinghai was only 0.3 × 10^9^ m³, while the tertiary industry water consumption in Guangdong reached 10.79 × 10^9^ m³, in 2020.

### Analysis of the decomposition effects

**Figure 2:**
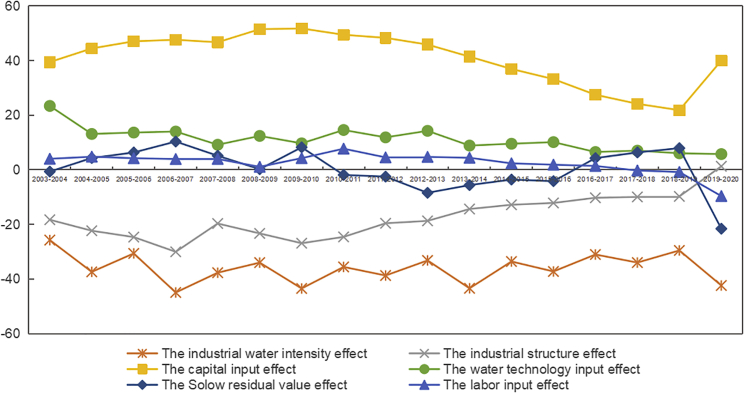
Decomposition factor effect of water consumption changes in China from 2003 to 2020

Six key driving factors of water consumption, the industrial water intensity effect, the industrial structure effect, the water technology input effect, the capital input effect, the labor input effect, and the Solow residual effect are decomposed by the LMDI model. The changing trend of each effect is shown in Figure 2. The industrial water intensity effect and the cumulative effect of the industrial structure effect are negative, while the capital input effect, the water technology input effect, the cumulative effect of the labor input effect, and the Solow residual value effect are positive. The industrial water intensity effect has the strongest water-saving effect, reaching −612.26 × 10^9^ m³, while the capital input effect leads to an increase of 697.02 × 10^9^ m³.

**Figure 3:**
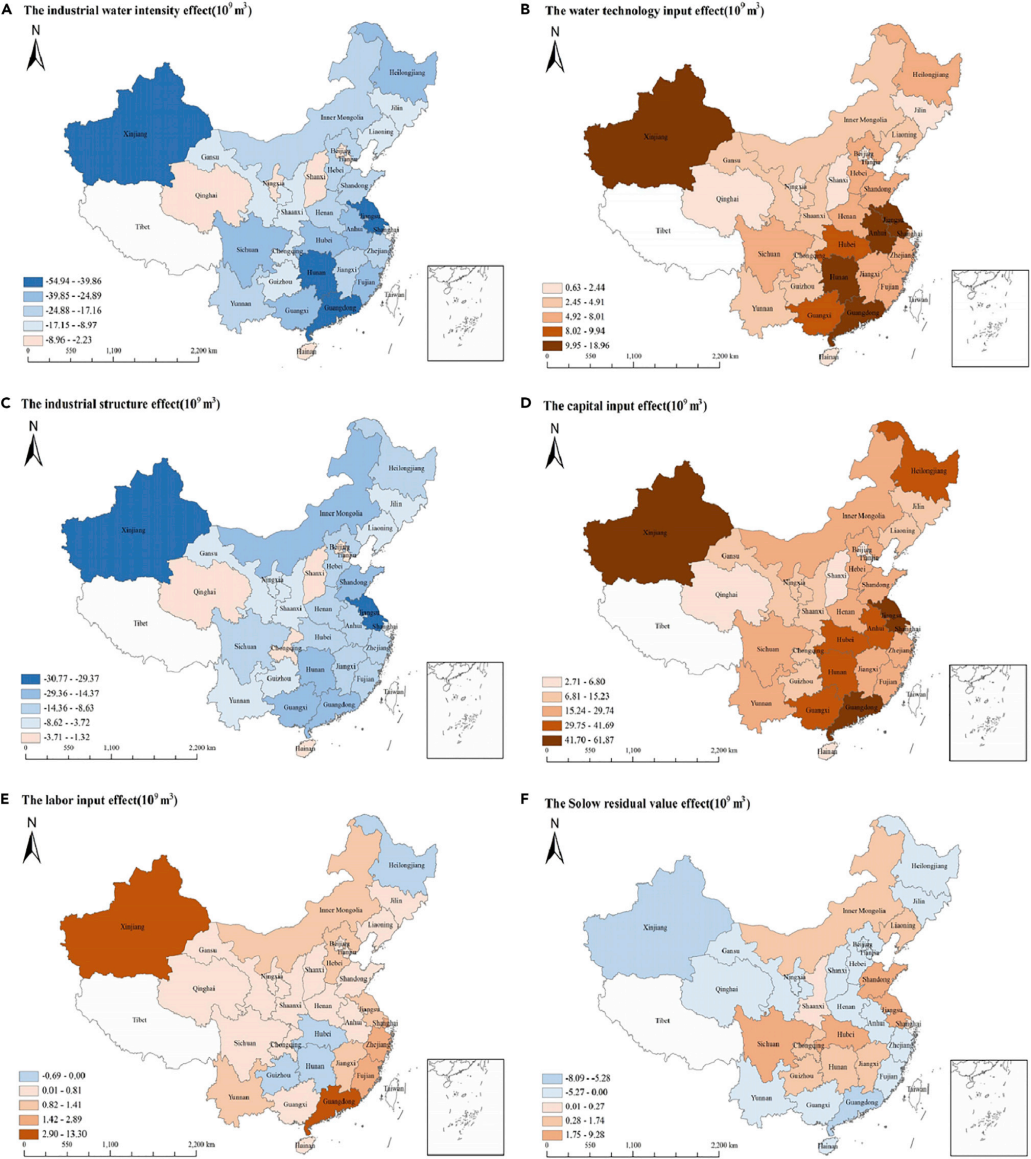
Distribution of driving effects of water consumption changes in China’s provinces (A) The intensity effect. (B) The scale effect. (C) The industry structure effect. (D) The capital input effect. (E) The labor input effect. (F) The Solow residual value effect.

The geographical distribution of the driving effects for each factor and total water consumption is shown in Figure 3. For further analysis, we divide the 30 provinces into northeast, eastern, central, and western regions. In order to make the driving effects in each region comparable, driving factor effects of unit water consumption, calculated by dividing by water consumption, are used in this article.

### Impact of water technological progress on China’s water consumption

Based on the model combining the C-D production function and the LMDI, the industrial water intensity effect and the water technology input effect are calculated to identify the intensity effect and scale effect of water technological progress on water consumption accurately.

### *The intensity effect of water technological progress inhibits water consumption*

China is in the stage of economic transformation in sustainable development paths and supporting the development of high-tech industries. The intensity effect of technology change reduces water consumption by decreasing the industrial water intensity. The cumulative contribution of this effect to water consumption has reached −612.26 × 10^9^ m³. From 2003 to 2020, the inhibitory effect of industrial water intensity effect fluctuated considerably, as shown in Figure 2. During 2003–2007, the contribution of this effect increased from −25.74 × 10^9^ m³ to −44.92 × 10^9^ m³. From 2007 to 2019, this effect’s contribution tended to a fluctuating downward trend until it increased again in 2020 to −42.36 × 10^9^ m³.

**Figure 4:**
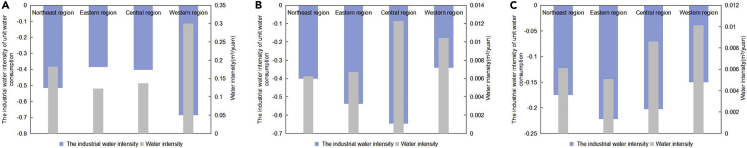
Industrial differences in water intensity and the industrial water intensity effects unit water consumption in the four major regions from 2003 to 2020 (for divisions of four regions see Table S1) (A) The primary industry. (B) The secondary industry. (C) The tertiary industry.

From a regional perspective, the industrial water intensity effect led to a reduction in water consumption in all regions, with the most significant inhibitory effect in the central region while the least in the northeast region, as shown in Figures 3A and 4. The industrial water intensity effect is related to the regional economic development mode and industrial water intensity. Figure 4 shows the industrial differences in water intensity and the industrial water intensity effects of unit water consumption in the four regions. The eastern region focused on developing a high-quality manufacturing industry and service industry with a high-level water technology, so the water intensity of the secondary and tertiary industries had a significant inhibitory effect on water consumption. In the central region, the water intensity of the secondary industry was the highest, but with the improvement of water technology level, its water-saving effect was the most significant. Both the northeast and western regions are important grain bases, with high water intensity in the primary industry. The average water intensity of the primary industry reached 0.2 m³/yuan, which was 24 times larger than that of the tertiary industry. In the future, it is necessary to reduce the water intensity of the primary industry, especially in the northeast and western regions.

### *The scale effect of water technological progress increases water consumption*

**Figure 5:**
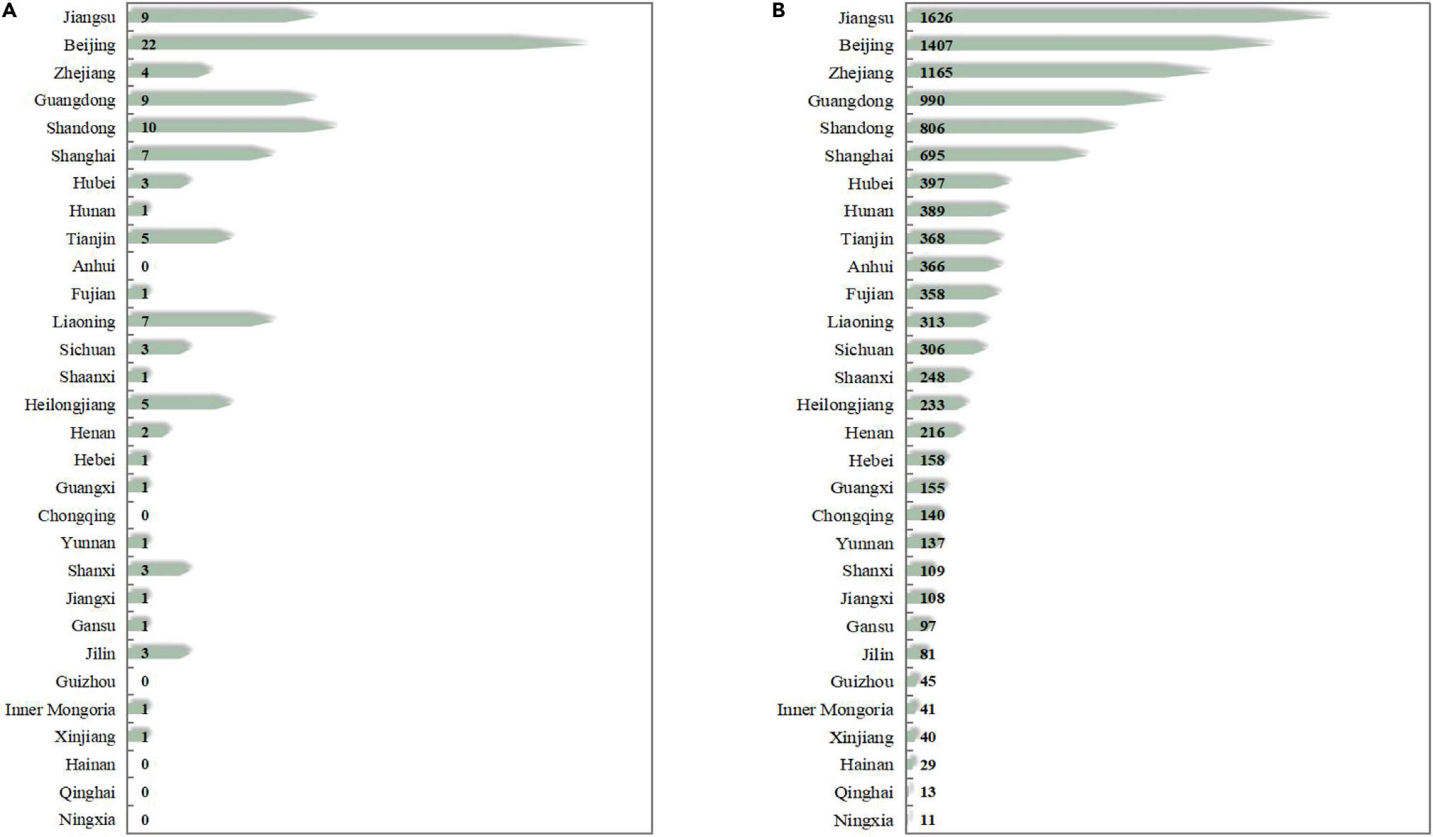
Cumulative patent authorization of water technologies in 2003 and 2020 (A) Cumulative patent authorization of water technologies in 2003. (B) Cumulative patent authorization of water technologies in 2020.

Water technological progress brings about economic growth, leading to an increase in water consumption. In this study, the water technology input effect is calculated to measure the scale effect of water technology progress on economic development. Figure 5 shows cumulative patent authorization of water technologies. From 2003 to 2020, the cumulative number of patent licenses has increased by 106 times, and by 2020, the number of patent licenses has reached 11,047 in China.

As shown in Figure 2, the water technology input effect significantly promotes the increase in water consumption, reaching 189.91 × 10^9^ m³. From a year-by-year perspective, the water technology input effect showed a trend of fluctuating downward, which indicated that the scale effect has gradually converged. From 2003 to 2004, the water technology input effect was 23.38 × 10^9^ m³, while from 2019 to 2020, it declined to 5.75 × 10^9^ m³. This can be explained by the increase in the marginal cost of water technology innovation. Repeated R&D investment and ineffective investment increased at a later stage, so that the cost of water technology increased and the contribution to economic expansion declined in the process of economic development.

From a regional perspective, we can find significant differences in the water technology input effect in different regions, as shown in Figure 3B. Water technology change had the most notable positive value of scale effect on water consumption for high-income regions, with a unit effect of 0.44 and 0.36 in central and eastern regions. The water technology level and economic development level of the eastern region are higher than those in the central region, while the water technology effect is not the case. This is related to the stage and orientation of water technology innovation. The central region is the key area for China’s new round of industrialization and urbanization, with a high level of economic development in recent years. In 2020, the number of water technology patents in the central and eastern regions accounted for 83.16% of the total, and GDP accounted for 72.87% of the total. This indicated that the water technology level in the central and eastern regions was higher than that in the northeastern and western regions, and technological progress promotes economic expansion. In the northeast and western regions, the economic development is mainly based on the factor-dependent model. Furthermore, during the 12th Five-Year Plan period, the National Development and Re-form Commission clearly stated that it was forbidden to transfer high water-consuming projects to the western region. For these reasons, the scale effect brought by water technological progress in northeast and western regions was relatively small.

### *Comprehensive effect of water technological progress inhibits water consumption*

**Figure 6:**
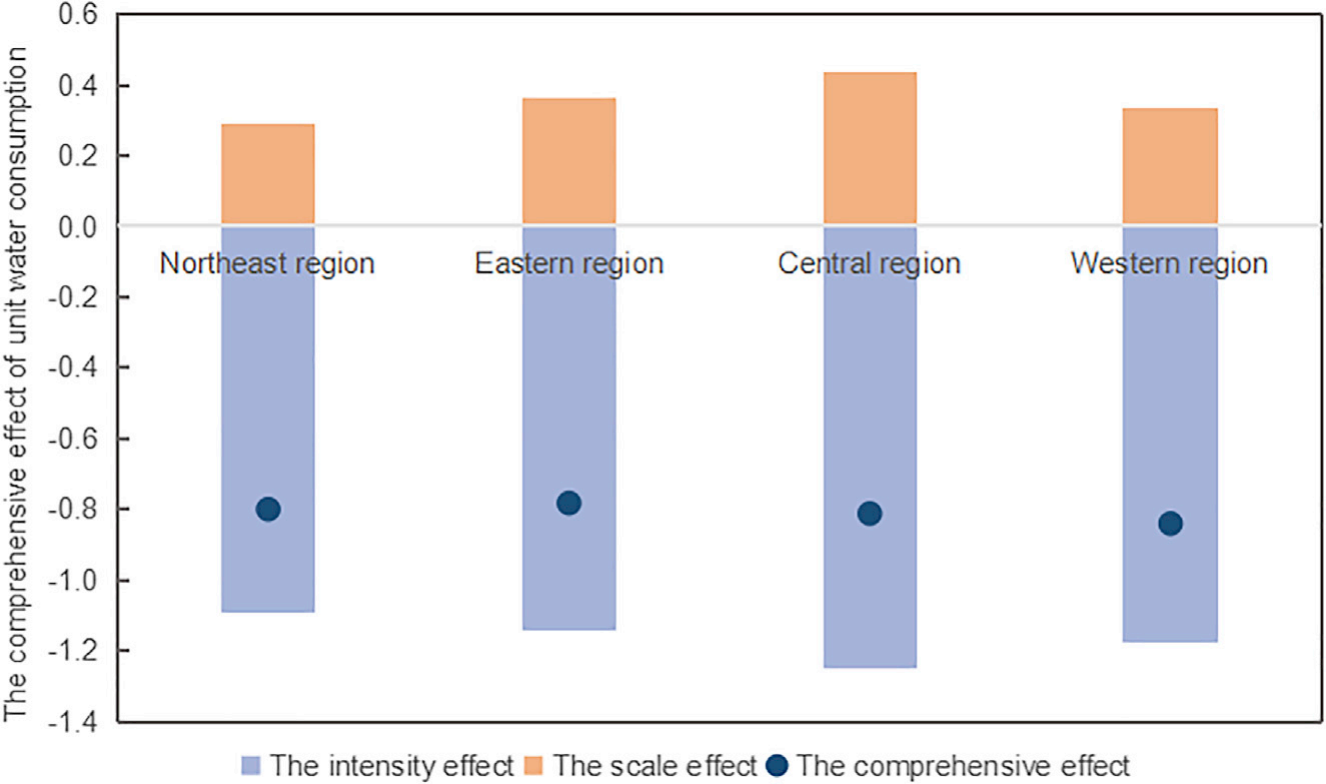
The comprehensive effect of technological progress on China’s water consumption changes from 2003 to 2020 (for driving factor effects of unit water consumption see Table S2)

Negative values of intensity effect are greater than positive values of scale effect of water technological progress. As shown in Figure 6, the ratio of the scale effect of water technological progress to the intensity effect was 31% in China. The dual effect gap of technology among regions was obvious. For example, in Jilin and Inner Mongolia, the ratio was only 21.42% and 24.74%, while the absolute value ratio of Jiangsu and Anhui could reach 41.87% and 52.91%.

As shown in Table 1, this article establishes the business as usual (BAU) scenario and the no water technology change (NWT) scenario, based on the historical water consumption and the effectiveness of the water technological progress. Water technological progress contributed to a total reduction of 422.35 × 10^9^ m³ in national water consumption from 2003 to 2020. COVID-19 had a significant impact on China’s water consumption trends. In 2020, the water consumption under NWT increased by 6.7% compared with that under BAU for the lack of the water-saving effect of water technological progress. Among the four major regions, if under NWT scenario, the water consumption would have been 38 × 10^9^ m³, 146.48 × 10^9^ m³, 93.63 × 10^9^ m³, and 144.23 × 10^9^ m³ more than realistic water consumption in the northeast, eastern, central, and western regions.

**Table 1:** Potential water consumption under BAU and NWT scenarios (10^9^ m³)

year	Northeast region		Eastern region		Central region		Western region		China	
	BAU	NWT	BAU	NWT	BAU	NWT	BAU	NWT	BAU	NWT
2003	47.51	47.51	187.19	187.19	115.30	115.30	171.60	171.60	521.61	521.61
2004	48.46	51.48	197.42	197.11	122.74	117.62	175.14	179.90	543.76	546.11
2005	49.66	51.55	197.83	208.27	124.13	130.06	179.09	185.09	550.71	574.97
2006	52.61	52.29	201.87	208.85	130.99	132.54	181.23	189.99	566.69	583.67
2007	53.00	55.32	202.93	213.82	130.54	138.26	181.15	191.14	567.62	598.55
2008	53.65	57.12	202.07	213.84	136.37	140.49	183.13	192.29	575.23	603.74
2009	56.02	56.51	202.14	211.26	140.88	144.99	184.10	191.95	583.13	604.71
2010	57.98	59.76	203.47	213.96	141.49	150.87	183.75	195.88	586.70	620.47
2011	60.98	61.41	204.01	212.03	146.14	150.45	185.28	193.51	596.41	617.40
2012	61.45	64.35	200.30	212.60	145.19	153.89	193.38	196.31	600.32	627.15
2013	62.40	63.93	202.36	208.08	148.06	152.24	191.99	199.49	604.81	623.74
2014	62.91	64.98	201.48	210.21	141.37	154.67	190.37	200.78	596.12	630.64
2015	61.39	65.24	197.60	208.64	144.05	146.62	191.93	198.52	594.97	619.01
2016	60.61	62.73	194.79	203.71	142.35	149.47	188.87	197.79	586.62	613.71
2017	59.91	62.88	194.89	201.60	142.90	148.22	187.30	196.81	585.00	609.50
2018	58.02	61.51	192.21	201.28	143.83	148.38	184.27	194.12	578.33	605.30
2019	54.26	59.18	191.73	197.53	143.15	148.86	184.86	191.91	574.00	597.48
2020	54.00	55.09	183.79	194.58	133.65	143.81	176.00	190.57	547.44	584.05

### Comparison of the effects between water technological progress and industrial structure

**Figure 7:**
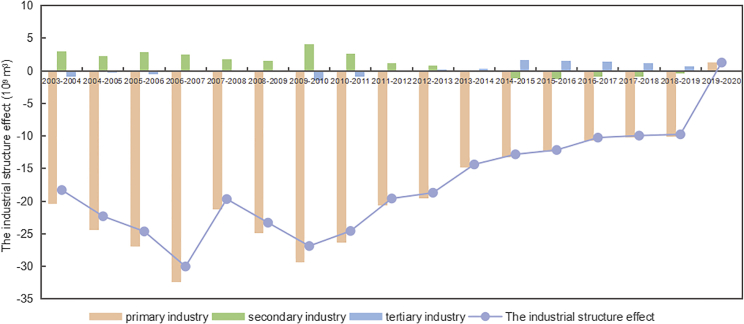
Industrial structure effect of three industries on China’s water consumption changes from 2003 to 2020

The industrial structure effect reveals the contribution of industrial structure changes in water consumption changes. The trends of it and the differences within the three industries during 2003-2020 are shown in Figure 7. The optimization and upgrading of the industrial structure reduced water consumption by 296.02 × 10^9^ m³. From the perspective of industrial differences, the water-saving effect of the primary industry reached −316.49 × 10^9^ m³, while the industrial structure effects of the secondary and tertiary industries both led to an increase in water consumption. After 2013, the contribution direction of the secondary industry and the tertiary industry underwent opposite changes. The reason was that China entered the new normal economic development stage, and the tertiary industry gradually became an important pillar industry for China’s economic development. From a regional perspective, the distribution of industrial structure effects in different regions is shown in Figure 3C. The central and western regions had the most significant unit industrial structure effects, reaching −0.599 and −0.596.

**Figure 8:**
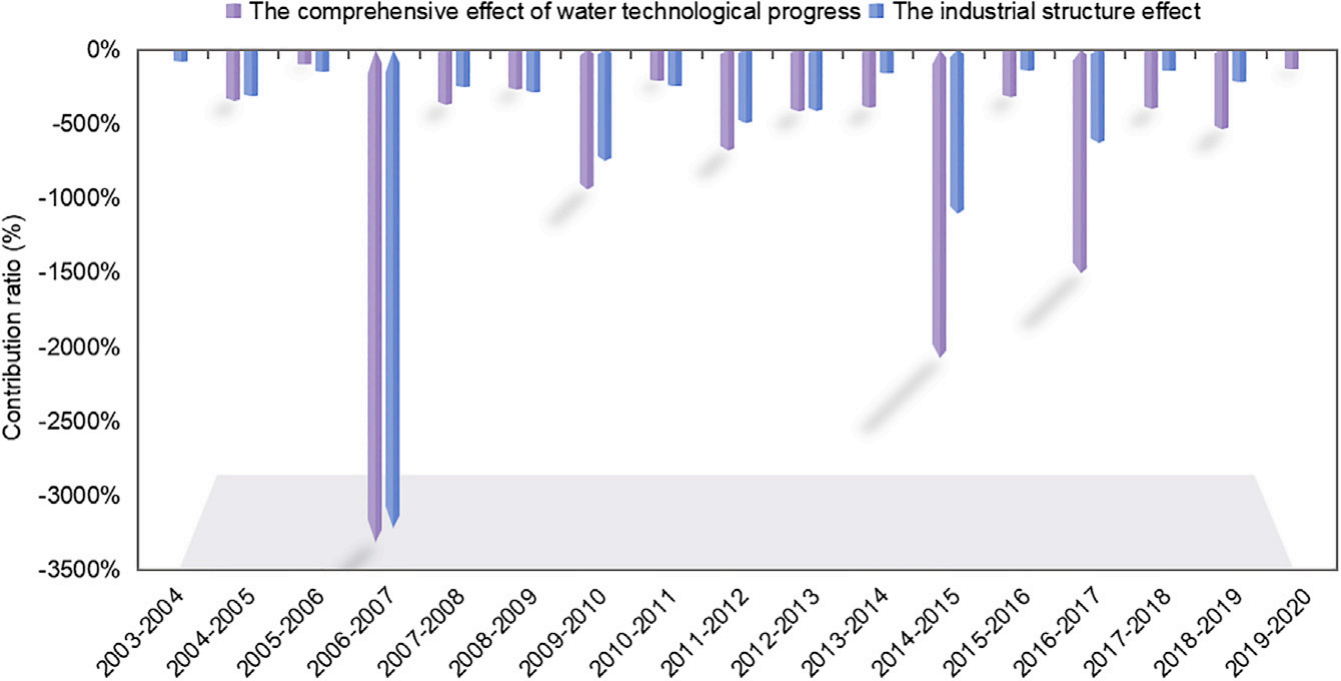
Contribution ratio of the comprehensive effect of water technological progress and the industrial structure effect The contribution ratio is the ratio of the comprehensive effect of water technological progress and the industrial structure effect to the water consumption change.

The industrial structure effect was second to the comprehensive effect of water technological progress, a difference of 126.33 × 10^9^ m³. Figure 8 shows the contribution ratio of the comprehensive effect of water technological progress and the industrial structure effect. Compared with the industrial structure, the contribution of water technology progress to the water-saving effect showed an increasing trend.

### Impact of factor input and the Solow Residual on water consumption

#### Factor input effect increases water consumption

Factor input (capital input and labor input) effect reveals the extent to which the changes in capital input and labor input affect water consumption changes. Factor input resulted in a cumulative increase of 739.54 × 10^9^ m³ of water consumption. The contribution of capital input was much higher than labor input, reaching 94.25%. From a regional perspective, owing to different economic levels, there were differences in the regional factors’ input effects (Figures 3D and 3E). The factor input in the central regions was the most active, contributing 1.53 m³ to unit water consumption. Among all the provinces, the capital input effect in Guangdong and Hunan reached 50.79 × 10^9^ m³ and 41.69 × 10^9^ m³. The unit labor input effect of the eastern region was larger than other regions, reaching 0.11, because the eastern region had ample employment opportunities and was a main population migration area.

#### The Solow residual effect increases water consumption

The Solow residual value is the “residual value” that the contribution of capital, labor, and water technology in the process of economic growth cannot explain. As shown in Figure 2, the Solow Residual effect made opposite contributions to water consumption in different years, and the cumulative contribution was positive, which was 4.66 × 10^9^ m³. From a regional perspective, we can find significant differences in four regions, as shown in Figure 3F. The Solow residual value effect closely relates to the regional technology level. The Solow residual value effect in the central and eastern regions was positive, while this effect in the northeast and western regions was negative.

## Discussions

Water shortage is a great challenge to meet an increasing demand for human survival and sustainable economic development in this century (Brauman et al., 2016). Research on the relationship between water technological progress and water consumption has emphasized the inhibitory effect of water technological progress (Li et al., 2021) but ignored its positive role of it in increasing water consumption by increasing the scale of economic output. With the acceleration of industrialization and the rapid expansion of economic scale, China is one of the countries with serious water shortage problems. This article takes China as the case to analyze the driving factors of water consumption from the perspective of temporal and spatial differences, with particular attention given to the dual effects of water technological progress. It’s valuable to explore a feasible way of water conservation in the consideration of sustainable development and regional difference.

The contribution of water technological progress to water consumption reduction was overestimated in the previous studies. The findings of this article indicated that water technological progress reduced water consumption in general, while the scale effect increased water consumption and offset part of the water-saving effect of the intensity effect. The advancement of water technology promotes an increase in economic scale, which leads to an increase in water consumption. From 2003 to 2020, the water-saving effect of water technological progress was overestimated by 44.97% in China. In this case, countries should not blindly emphasize reducing water consumption depending on water technology but should pay attention to other water-saving measures to achieve a win-win between economic growth and water efficiency improvement. Please note that we just emphasize that countries should mitigate the trade-off of water technological progress rather than denying the important contribution of technological progress to water conservation.

To mitigate the trade-off of water technological progress and alleviate the water shortage pressure, countries should strengthen technology guidance and diffusion, improve the water trading market under government regulation and adjust the industrial structure. (1) The water-saving effect of technological progress varies greatly in different regions. A unified national water technology trading market should be established to promote the diffusion and implementation of water technology in backward regions. Among three industries, the agricultural sector consumes most water resources with the minimum share of economic output. To enhance the intensity effect of water technological progress, it is essential to promote agricultural water-saving irrigation technologies such as sprinkler irrigation, micro-irrigation, drip irrigation, low-pressure pipeline irrigation, and channel anti-seepage to improve agricultural water use efficiency. (2) The scale effect of water technological progress increases water consumption. Market is an important tool for reducing the negative resource effects of water technology. Water price regulation and water rights trading based on the water market can increase the economic cost of water use by enterprises and realize the optimal allocation of water resources. To prevent the expansion of high-margin products with excessive water consumption, the water market should be established under strict government management. (3) Our findings show that the industrial structure effect inhibits water consumption, second to the comprehensive effect of water technological progress. However, the industrial structure effect has declined in recent years because the process of industrial restructuring has slowed down. Under the constraints of economic development and water conservation, the industrial structure adjustment faces greater challenges. The economic development model should be changed from relying on resource consumption to relying on scientific and technological progress, improvement of labor quality, and management innovation. Taking China as an example, the government should advance the modernization and transformation of the agricultural sector, and moderately limit the proportion of high water-consuming sectors such as metal ore mining and dressing sector, food and tobacco sector, textile sector, coking and nuclear fuel processing sector, and non-metallic mineral manufacturing sector. At the same time, it is necessary to encourage the development of sectors with cleaner production such as transportation equipment manufacturing, electronic equipment manufacturing, information transmission, software, and information technology services.

The effect of water technological progress has obvious regional disparity. In order to alleviate the water shortage problem in different regions, it is necessary to implement water-saving policies tailored to local conditions and explore differentiated water-saving paths. The northeast region is the important commodity grain base and old industrial base in China, with a minimum level of water-saving effect of water technological progress. Upgrading of agricultural water-saving technologies should be strengthened, and the water-saving crops should be promoted to reduce the proportion of water consumption in agriculture. The green transformation of the industry should be accelerated in the northeast region, especially focusing on the energy sector. The eastern region has a large number of professionals, advanced technology, and abundant funds. The government should rely on the existing resources to further promote the development of the service industry and realize upgrading of the industrial structure. In addition, as a region with mature technology level, it is necessary to promote the pilot of water rights trading in other cities in the future, referring to Guangdong and Nanjing. The central region is the main resources and raw materials supply base, where Shanxi is rich in coal resources, while Hunan, Hubei, Henan, and Jiangxi are important grain-producing areas. The central region has a high level of water-saving effect owing to water technological progress. It has also undertaken the industrial transfer from the eastern region. The access threshold with resource constraints should be raised, strictly controlling the transfer of high water-consuming sectors such as textile and garment manufacturing and non-metallic mineral product. The western region is rich in water resources to be developed but with high water intensity. It’s helpful for the western region to accept technological diffusion from the eastern region. In addition, the service industry should be developed with the advantages of regional natural endowments to realize decoupling between economic development and inefficient resource use. Based on regional disparity, it is necessary to break the shackles of resource flow between regions, and further promote the rational flow of human, material, capital, technology, experience, and other elements. In this way, it can be achieved to minimize the additional resource consumption of the technology and exert its water-saving effect.

The widespread COVID-19 pandemic has been leading to significant changes in various aspects globally, of which water has been facing new challenges as a basic environmental element (Jia et al., 2022). Taking China as an example, China’s water consumption dropped sharply to 547.44 × 10^9^ m³ in 2020. The article forecasts water consumption without a COVID-19 pandemic in 2020, based on the historical data from 2003 to 2019. The results show that the outbreak of a COVID-19 pandemic brought about an abnormal drop in water consumption, reaching 15.04 × 10^9^ m³, and an increase of the water-saving effect of water technological progress by 3.44 × 10^9^ m³. This is consistent with the findings revealed by other studies. However, these short-term positive changes are unsustainable, so it is necessary to promote technological progress to prepare for a rebound in water consumption during the recovery period.

### Limitations of the study

This article reveals the dual effects of water technological progress on water consumption and provides suggestions to mitigate the trade-off of water technological progress and regional disparity. There is a need to measure the dual effects of water technological progress in specific sectors and provide differentiated water-saving policies for different sectors. In addition, to provide a reference for future water resources planning, it’s valuable to predict the water-saving effects of water technological development in the future.

## STAR★Methods

### Key resources table

**Table:** 

REAGENT or RESOURCE	SOURCE	IDENTIFIER
**Deposited data**		

China’s provincial water consumption annual data	National Bureau of Statistics of China (NBSC)	http://data.stats.gov.cn/english/
China’s provincial GDP annual data	National Bureau of Statistics of China (NBSC)	http://data.stats.gov.cn/english/
China’s provincial gross fixed capital formation annual data	National Bureau of Statistics of China (NBSC)	http://data.stats.gov.cn/english/
China’s provincial price index for investment in fixed assets annual data	National Bureau of Statistics of China (NBSC)	http://data.stats.gov.cn/english/
China’s provincial number of employed persons annual data	National Bureau of Statistics of China (NBSC)	http://data.stats.gov.cn/english/
China’s provincial patent data for water technologies annual data	Qizhdao Patent Database	https://www.qizhidao.com
Raw data of of the LMDI model	Mendeley Data	https://data.mendeley.com/datasets/r2jt2pgvcf/1

**Software and algorithms**		

Stata	Statistical software for data science	https://www.stata.com/

### Resource availability

#### Lead contact

Further information and requests for resources should be directed to and will be fulfilled by the Lead Contact, Pingjiang (jiangping@fudan.edu.cn).

#### Materials availability

This study did not generate new unique reagents.

### Method details

#### Kaya identities of C-D production function extension

Water technological progress is one of the main driving factors of water consumption. This paper decomposes the influencing factors of water consumption to analyze the changes in China’s water consumption from the perspective of water technological progress. Regarding the factor decomposition in the field of resource consumption and carbon emissions, Yoyichi Kaya (1989) proposed the Kaya identity in the early 20th century. The Kaya identity decomposed carbon emissions into factors including energy efficiency, energy structure, economic level, and population scale. According to Kaya identities and Johan’s expansion formula (Johan et al., 2002), the total water consumption can be decomposed as follows:

(Equation 2) $$TW^{\text{t}}=\sum_{i}\sum_{j}\left(W_{\text{ij}}^{\text{t}}/V_{\text{ij}}^{\text{t}}\right)\cdot \left(V_{\text{ij}}^{\text{t}}/G_{\text{i}}^{\text{t}}\right)\cdot G_{\text{i}}^{\text{t}}=\sum_{i}\sum_{j}WOV_{\text{ij}}^{\text{t}}\cdot VOG_{\text{ij}}^{\text{t}}\cdot GDP_{\text{i}}^{\text{t}}$$

where *i* denotes the province; *j* denotes the industry; *TW*_*t*_ denotes the total water consumption in period *t*; *W*_*ij*_ denotes the water consumption of the j-th industry in the i-th province; *V*_*ij*_ denotes the actual output value of the j-th industry in the i-th province; and *G*_*i*_ denotes the gross domestic product (GDP) of the i-th province. $WOV_{ij}^{t}=W_{\text{ij}}^{\text{t}}/V_{\text{ij}}^{\text{t}}$ denotes the ratio of industrial water consumption to industrial output value, which is defined as the water intensity effect; $VOG_{i\text{j}}^{t}=V_{\text{ij}}^{\text{t}}/G_{\text{i}}^{\text{t}}$ denotes the ratio of the actual output value of the j-th industry to GDP, which is defined as the industrial structure effect; $GDP_{i}^{t}$ denotes the GDP of the i-th province in period t, which is defined as the economic development effect.

Capital, labor and technology are the basic factors affecting economic growth. The C-D production function studies the relationship between the input and output of each factor and is widely used in economic quantitative analysis. However, the traditional C-D production function fails to distinguish contribution of water technology to economic growth from other technologies. To further explore how water technological progress affects China's water consumption by promoting economic growth, this study constructs an extended C-D production function with water technology separated as an independent variable *WT*. The extended C-D production function is shown as below:

(Equation 3) $$GDP^{\text{t}}=A^{\text{t}}\cdot {\left(K^{\text{t}}\right)}^{\alpha }\cdot {\left(L^{\text{t}}\right)}^{\beta }\cdot {\left(WT^{\text{t}}\right)}^{\gamma }$$

Further, this paper embeds the extened C-D production function into the kaya identity as follow:

(Equation 4) $$TW^{\text{t}}=\sum_{i}\sum_{j}WOV_{\text{ij}}^{\text{t}}\cdot VOG_{\text{ij}}^{\text{t}}\cdot A^{\text{t}}\cdot {\left(K^{\text{t}}\right)}^{\alpha }\cdot {\left(L^{\text{t}}\right)}^{\beta }\cdot {\left(WT^{\text{t}}\right)}^{\gamma }$$

where *A*_*t*_ denotes the Solow residual value effect; (*K*^*t*^)^*α*^ denotes the capital input effect; (*L*^*t*^)^*β*^ denotes the labor input effect; (*WT*^*t*^)^*γ*^ denotes the water technology input effect.

Water technological progress has a dual impact on water consumption. Among the above influencing factors, industrial water intensity denotes the water consumption per unit output value, which is mainly affected by technological progress. Therefore, the industrial water intensity effect $WOV_{ij}^{t}$ is regarded as the intensity effect of water technological progress. The water technology input denotes the contribution of water technology progress to economic growth. Therefore, the water technology input effect *(WT*^*t*^)^*γ*^ is regarded as the scale effect of technological progress.

The calculation process of the extended C-D production function is as follows:

(Equation 5) $$\left\{ \begin{array}{c} GDP^{\text{t}}=A^{\text{t}}\cdot {\left(K^{\text{t}}\right)}^{\alpha }\cdot {\left(L^{\text{t}}\right)}^{\beta }\cdot {\left(WT^{\text{t}}\right)}^{\gamma }\\ \alpha +\beta +\gamma =1\\ A,\alpha ,\beta ,\gamma >0 \end{array}\right.$$

where *α*, *β* and *γ* denote capital elasticity, labor elasticity and water technology elasticity.

*α*, *β* and *γ* are respectively equal to the capital share and labor share in the GDP. Using the least square method, it can be calculated that α = 0.541, β = 0.391, γ = 0.068. And the Solow residual value of different provinces and different years can be calculated by the following formula:

(Equation 6) $$A=Y/\left[{\left(L\right)}^{\alpha }{\left(K\right)}^{\beta }{\left(WT\right)}^{\gamma }\right]$$

#### LMDI decomposition model

There are two methods used to decompose index changes: Structural Decomposition Analysis (SDA) and Index Decomposition Analysis (IDA). The LMDI method proposed by Ang (2005) belongs to the IDA methods. This method can decompose the contribution of each factor to the total change and has the advantages of easy decomposition and no residual (Ang, 2015). The LMDI method can be divided into two calculation types called the addition model and multiplication model. The addition model, which is better suited for quantity index than intensity index. Water consumption is a quantity index, so this paper uses the LMDI addition model to decompose the driving factors of China’s water consumption. The total amount of water consumption changes (ΔW) in the total time span [0, t] can be decomposed into six driving factors: the industrial water intensity effect (ΔW_wv_), the industrial structure effect (ΔW_vg_), the capital input effect (ΔW_k_), the labor input effect (ΔW_l_), the water technology input effect (ΔW_wt_) and the Solow residual value effect (ΔW_a_). The results of decomposition can be expressed as follows:

(Equation 7) $$\text{Δ}W^{\text{t}}=\text{Δ}W_{wv}^{\text{t}}+\text{Δ}W_{vg}^{\text{t}}+\text{Δ}W_{k}^{\text{t}}+\text{Δ}W_{l}^{\text{t}}+\text{Δ}W_{wt}^{\text{t}}+\text{Δ}W_{a}^{\text{t}}$$

The effects of six driving factors can be calculated as follows in Equations 8, 9, 10, 11, 12, and 13:

(Equation 8) $$\text{Δ}W_{\text{wv}}^{t}=\sum_{\text{i}}\sum_{\text{j}}\left(\frac{W_{\text{ij}}^{t}-W_{\text{ij}}^{t-1}}{\ln\;W_{\text{ij}}^{t}-\ln\;W_{\text{ij}}^{t-1}}\times \ln\frac{WOV_{ij}^{t}}{WOV_{\text{ij}}^{t-1}}\right)$$

(Equation 9) $$\text{Δ}W_{\text{vg}}^{t}=\sum_{\text{i}}\sum_{\text{j}}\left(\frac{W_{\text{ij}}^{t}-W_{\text{ij}}^{t-1}}{\ln\;W_{\text{ij}}^{t}-\ln\;W_{\text{ij}}^{t-1}}\times \ln\frac{VOG_{ij}^{t}}{VOG_{\text{ij}}^{t-1}}\right)$$

(Equation 10) $$\text{Δ}W_{\text{k}}^{t}=\sum_{\text{i}}\sum_{\text{j}}\left(\frac{W_{\text{ij}}^{t}-W_{\text{ij}}^{t-1}}{\ln\;W_{\text{ij}}^{t}-\ln\;W_{\text{ij}}^{t-1}}\times \ln\frac{{\left(K_{i}^{t}\right)}^{\alpha }}{{\left(K_{i}^{t-1}\right)}^{\alpha }}\right)$$

(Equation 11) $$\text{Δ}W_{\text{l}}^{t}=\sum_{\text{i}}\sum_{\text{j}}\left(\frac{W_{\text{ij}}^{t}-W_{\text{ij}}^{t-1}}{\ln\;W_{\text{ij}}^{t}-\ln\;W_{\text{ij}}^{t-1}}\times \ln\frac{{\left(L_{i}^{t}\right)}^{\beta }}{{\left(L_{i}^{t-1}\right)}^{\beta }}\right)$$

(Equation 12) $$\text{Δ}W_{\text{wt}}^{t}=\sum_{\text{i}}\sum_{\text{j}}\left(\frac{W_{\text{ij}}^{t}-W_{\text{ij}}^{t-1}}{\ln\;W_{\text{ij}}^{t}-\ln\;W_{\text{ij}}^{t-1}}\times \ln\frac{{\left(WT_{i}^{t}\right)}^{\gamma }}{{\left(WT_{i}^{t-1}\right)}^{\gamma }}\right)$$

(Equation 13) $$\text{Δ}W_{\text{a}}^{t}=\sum_{\text{i}}\sum_{\text{j}}\left(\frac{W_{\text{ij}}^{t}-W_{\text{ij}}^{t-1}}{\ln\;W_{\text{ij}}^{t}-\ln\;W_{\text{ij}}^{t-1}}\times \ln\frac{A_{i}^{t}}{A_{i}^{t-1}}\right)$$

## Data Availability

The capital stock is calculated using the perpetual inventory method, as shown in Equation (1):(Equation 1)$${\text{K}}_{\text{i}}^{\text{t}}={\text{K}}_{i}^{t-1}\left(1-\text{δ}\right)+{\text{I}}_{\text{i}}^{t}/{\text{P}}^{\text{t}}$$where *i* denotes the i-th province; *t* denotes the year; $K_{i}^{t}$ and $K_{i}^{t-1}$ respectively denote the capital stock of the i-th province in period t and t-1; δ denotes the capital depreciation rate; $I_{i}^{t}$ denotes the nominal total investment of the i-th province in period *t*; *P*^*t*^ denotes the fixed asset investment price index in period *t*. The capital depreciation rate is 9.6%. As the indicators of water consumption in statistical data are not divided according to the three industries, but rather into agricultural water, industrial water, domestic water, and ecological water, in order to correspond with the three industries, we adjust the four types of water. Referring to the research of Zhang et al. (2020), we take agricultural water as primary industry water; industrial water as secondary industry water, and domestic water as tertiary water approximately. The total water consumption is obtained from the adjusted three types of industry water. This study did not generate any codes. The preliminary data are available on Mendeley Data: https://data.mendeley.com/datasets/r2jt2pgvcf/1.

## Supplemental information

Table S1. Four regions in China, related to Figure 4

Table S2. Driving effects in regions, related to Figure 6
